# Myocardin Overexpression Is Sufficient for Promoting the Development of a Mature Smooth Muscle Cell-Like Phenotype from Human Embryonic Stem Cells

**DOI:** 10.1371/journal.pone.0044052

**Published:** 2012-08-28

**Authors:** Linda Raphel, Amarnath Talasila, Christine Cheung, Sanjay Sinha

**Affiliations:** Anne McLaren Laboratory for Regenerative Medicine, University of Cambridge, Cambridge, United Kingdom; University of Bristol, United Kingdom

## Abstract

**Background:**

Myocardin is thought to have a key role in smooth muscle cell (SMC) development by acting on CArG-dependent genes. However, it is unclear whether myocardin-induced SMC maturation and increases in agonist-induced calcium signalling are also associated with increases in the expression of non-CArG-dependent SMC-specific genes. Moreover, it is unknown whether myocardin promotes SMC development from human embryonic stem cells.

**Methodology/Principal:**

Findings The effects of adenoviral-mediated myocardin overexpression on SMC development in human ESC-derived embryoid bodies were investigated using immunofluorescence, flow cytometry and real time RT-PCR. Myocardin overexpression from day 10 to day 28 of embryoid body differentiation increased the number of smooth muscle α-actin^+^ and smooth muscle myosin heavy chain^+^ SMC-like cells and increased carbachol-induced contractile function. However, myocardin was found to selectively regulate only CArG-dependent SMC-specific genes. Nevertheless, myocardin expression appeared to be sufficient to specify the SMC lineage.

**Conclusions/Significance:**

Myocardin increases the development and maturation of SMC-like cells from human embryonic stem cells despite not activating the full repertoire of SMC genes. These findings have implications for vascular tissue engineering and other applications requiring large numbers of functional SMCs.

## Introduction

Myocardin was originally identified as a serum response factor (SRF) co-factor expressed only in cardiomyocytes and smooth muscle cells (SMCs) [1]. Myocardin increases activation of SRF-dependent genes by forming a higher order complex with SRF and facilitating its association with its CArG box DNA binding domain. Transcriptional activation is promoted by a variety of mechanisms including myocardin’s own powerful transcriptional activation domain [1] as well as recruitment of histone acetyl transferases [2] and histone demethylases [3]. We and others have shown that myocardin was required for SMC specific gene expression, such that an increase in myocardin expression upregulated SMC genes while a dominant negative form or knock down of endogenous myocardin decreased SMC gene expression [4]–[6]. Subsequently, it was demonstrated that a myocardin null mouse died at mid-gestation and lacked SMC around the developing dorsal aorta [7]. These and other findings led some investigators to dub myocardin as a SMC ‘master-regulator’ [8]. However, further studies using myocardin null mouse embryonic stem cells (ESCs) differentiated in vitro or injected into wild-type blastocysts [9], [10] have demonstrated that some SMCs are able to develop in the absence of myocardin and that there is a tissue specific requirement for myocardin in different SMC regions.

Other studies have also investigated the role of myocardin in SMC development and whether overexpression of this transcription factor led to SMC lineage commitment. Yoshida and colleagues [11] demonstrated that forced expression of myocardin in a variety of cell lines only increased CArG-box dependent SMC genes, with no effect on non-CArG dependent SMC genes such as smoothelin-B, focal adhesion kinase-related nonkinase (FRNK) and aortic carboxypeptidase-like protein (ACLP) suggesting that myocardin was not able to direct the full repertoire of activity required for SMC development. The progenitor cells used in this study included the P19-derived A404 SMC progenitor cells and 10T1/2 cells. Notably, neither of these cells have been shown to exhibit contraction on assuming a SMC-like phenotype so it is unclear to what extent they represent normal developmental events. Murine ES cells were also transduced with myocardin. These can generate contractile SMCs on differentiation in an embryoid body [12], but were not differentiated using this protocol in the study by Yoshida and colleagues [11]. Rat aortic SMCs were also used although these cells generally lose their contractile properties rapidly in serum containing media. Consequently, it is interesting to speculate whether cells already primed for, or undergoing more physiological SMC differentiation would express a wider range of SMC markers in response to myocardin given that other key developmental regulators may already be present in the cells. In contrast, Long and colleagues [13] suggested that expression of myocardin alone in a BC3H1 myogenic precursor line was sufficient to induce a SMC-like contractile phenotype, in particular at the ultrastructural and functional levels. These latter authors felt that myocardin was indeed functioning as a SMC master regulator but did not specifically examine CArG versus non-CArG genes. It is therefore unclear whether myocardin increases SMC contractility and function despite failing to induce non-CArG containing genes or whether cell types that show increased contractility in response to myocardin also upregulate the full repertoire of SMC genes.

Although SMC development has been studied in a wide range of systems such as *Xenopus*, avian and mouse, the lack of suitable models has limited the analysis of SMC development in man. Human ESCs, however, offer the opportunity to determine whether developmental mechanisms established in other species also hold true in man. Although many molecular mechanisms and pathways are conserved across species, there are examples in which mouse and human systems diverge, such as the need for leukaemia inhibitory factor (LIF) to maintain pluripotency in mouse [14] but not human ESCs [15]. Moreover, efficient methods to derive SMCs from human ESCs may potentially be used in novel regenerative medicine applications such as regeneration of the vessel wall as part of a therapeutic revascularisation strategy for ischaemic tissues. In this context, myocardin overexpression may represent a strategy for inducing the SMC lineage and promoting SMC development with high efficiency.

We examined whether overexpression of myocardin was able to promote the formation of SMCs in differentiating human embryoid bodies, whether contractile function in response to a vaso-active agonist was affected and whether increased gene expression was confined to the subset of CArG-dependent genes.

## Results

### Development of SMC in Human Embryoid Bodies

**Figure 1:**
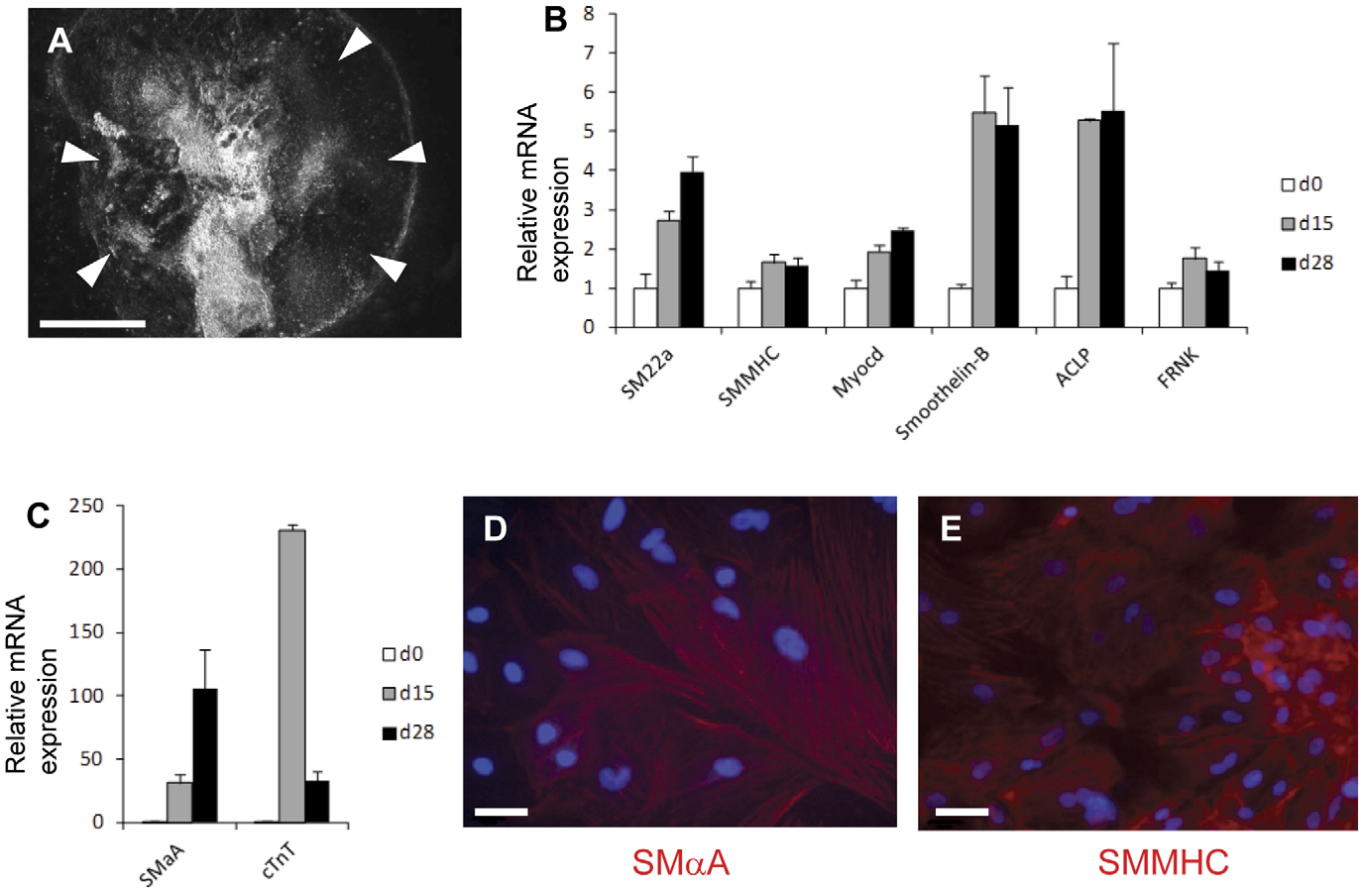
Development of SMCs in human embryoid bodies. (A) Low power dark field image of day 28 human embryoid body grown in 20% FBS showing outgrowth of cells (white arrowheads) from the central embryoid body mass. (B and C) Expression of SMC specific genes in embryoid bodies by real time RT-PCR at days 0, 15 and 28 is normalised by three housekeeping genes (GAPDH, UBC, 18S) and then presented relative to undifferentiated human ESCs. RT-PCR data represent means from three independent experiments. Bars represent s.e.m. Cells that stain for SMαA (D) and SMMHC (E) clearly seen at day 28 within embryoid bodies by immunofluorescence. Nuclei counterstained (blue) with DAPI. Bar in A = 1000 µm, bars in D and E = 20 µm.

Human ESC clumps were induced to undergo serum-induced differentiation in embryoid bodies initially in suspension then plated onto gelatin coated plates as described in the materials and methods. This resulted in a dense outgrowth of differentiating cells (marked by arrowheads in Fig. 1A) from the original embryoid body. RNA was extracted from undifferentiated H9 human ESCs (day 0) and from the embryoid bodies at days 15 and 28. Quantitative RT-PCR studies showed increase in the expression of a wide variety of SMC marker genes during differentiation (Fig. 1B). There was considerable variability in the degree of induction of key SMC marker genes; smooth muscle α-actin (SMαA) and the cardiac marker cardiac troponin T (cTnT) were highly expressed compared to the source hESC population (Fig. 1C), while relatively small increases in the expression of FRNK and the mature SMC marker, smooth muscle myosin heavy chain (SMMHC) were detected.

Immunocytochemistry for SMαA and SMMHC at day 28 revealed the presence of groups of cells that stained for SMC markers within the embryoid body outgrowth (Fig. 1D & E). Consistent with the low level of SMMHC mRNA expression, areas displaying SMMHC immunostaining were less frequent than areas with SMαA immunostaining suggesting that the majority of the SMCs in this system were developmentally immature. Under the differentiation conditions used in this experiment, we have previously seen spontaneous contraction of clusters of SMCs in mouse embryoid bodies at approximately day 20 [16]. However, spontaneous SMC-like contraction in human embryoid bodies was not detected unless these were cultured for 45 days or more (data not shown) suggesting a delay in SMC maturation in human embryoid bodies compared to mouse under the present conditions.

### Regulation of SMC Specific Markers and Cell Numbers in Human Embryoid Bodies by Myocardin

**Figure 2:**
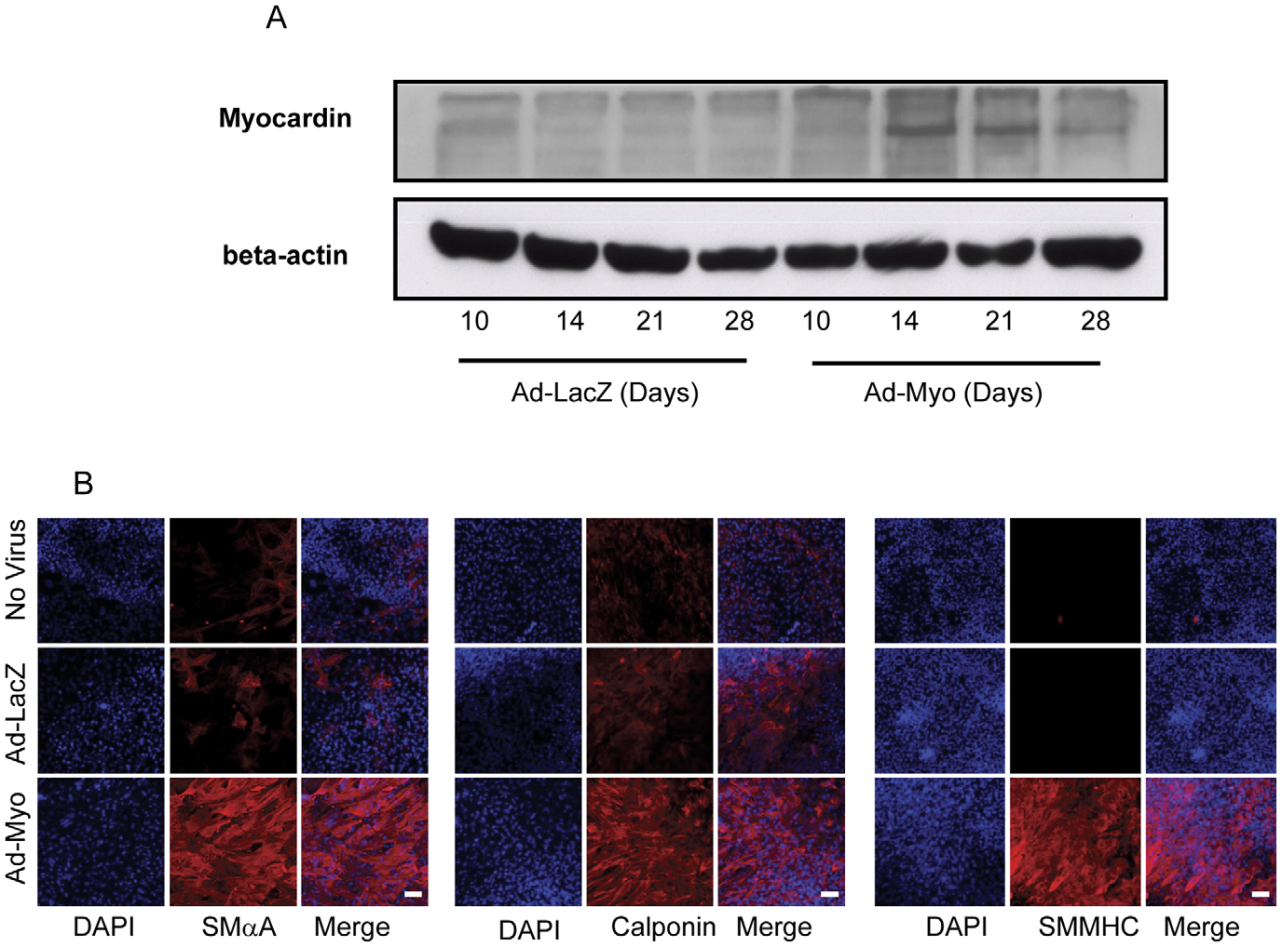
Myocardin overexpression increases SMαA, calponin and SMMHC staining. Immunoblotting of embryoid bodies transduced with Ad-LacZ or Ad-Myo confirm myocardin overexpression at a variety of developmental times (A). Immunofluorescent assessment of day 28 embryoid bodies reveals an increase in SMαA, calponin and SMMHC (B) staining following treatment with Ad-Myo from day 10 onwards, in comparison to no treatment or Ad-LacZ negative controls. Photomicrograph exposure times were kept constant across all three treatment groups and deliberately low enough to clearly show the large increases in fluorescent intensity in the Ad-Myo group. Nuclei counterstained (blue) with DAPI. Bar = 100 µm.

To investigate whether exogenous myocardin would promote SMC development, differentiating embryoid bodies were transduced with adenoviruses expressing myocardin (Ad-Myo) or a negative control, β-galactosidase (Ad-LacZ) at days 10, 14, 18 and 23, as described in the materials and methods. Since the embryoid body is a multi-layered structure, viral transduction efficiency was optimised using varying concentrations of Ad-LacZ; the optimal dose of virus was chosen as 0.5×10^7^ pfu/ml (Fig. S1). Myocardin overexpression was confirmed by immunoblotting of the Ad-Myo transduced embryoid bodies (Fig. 2A). Expression levels were lower at day 28 than at earlier time points, which may reflect higher transduction efficiencies when embryoid bodies are smaller as opposed to reduced adenovirus access to all cells in large densely packed and multi-layered mature embryoid bodies.

Nevertheless, treatment with Ad-Myo resulted in a characteristic change in morphology of some outgrowth regions in the embryoid bodies, such that the cells appeared more spindle shaped or ‘SMC-like’ (Fig. S2). Immunocytochemistry at day 28 revealed areas with increased immunostaining for the SMC markers, SMαA, SMMHC and calponin in response to myocardin overexpression (Fig. 2B). SMMHC is a marker for mature SMCs and raised the possibility that myocardin may promote the development of a more mature SMC population.

**Figure 3:**
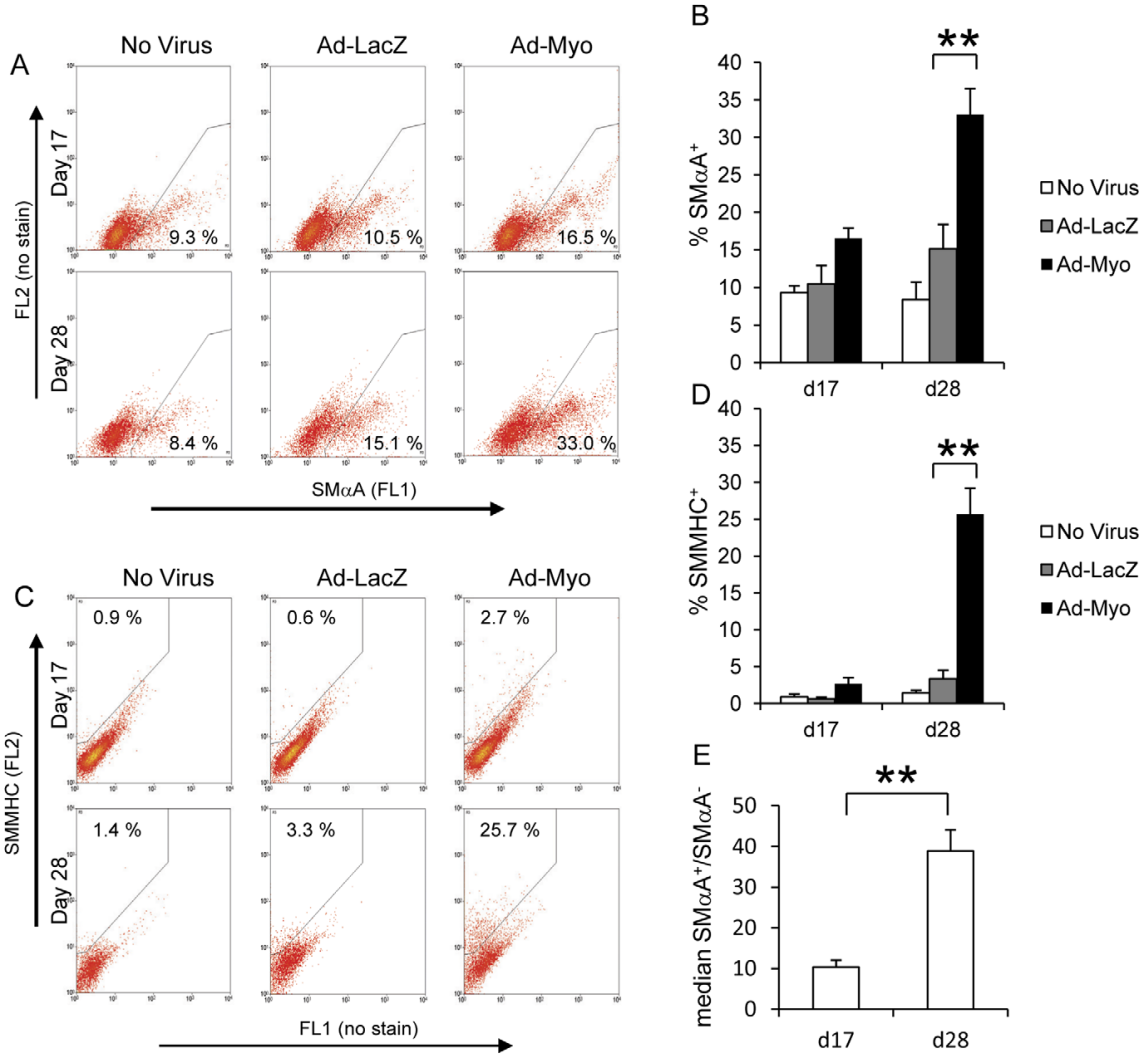
Myocardin overexpression increases number of SMC-like cells. Embryoid bodies were enzymatically dispersed into single cells at day 17 or day 28 and flow cytometric assessment for SMC markers was carried out. Groups that had been treated with no virus, Ad-LacZ or Ad-Myo from day 10 onwards were used to quantify the proportion of SMαA^+^ cells (A & B) and SMMHC^+^ cells (C & D). Both FL1 and FL2 channels were measured for all samples to distinguish specific signal for SMαA (FL1 in A) and SMMHC (FL2 in B) due to the high levels of autofluorescence in embryoid body-derived cells. In the no virus group, SMαA staining was quantified as median SMαA^+^ signal/median SMαA^−^ signal at both day 17 and day 28 (E). Data presented in A and C are representative flow cytometric plots from a single study with the means from three independent experiments specified in the gated regions and as bar charts ± s.e.m. (B, D & E). **p<0.01.

In order to quantify SMC numbers, we carried out flow cytometric analysis of SMαA and SMMHC expression on enzyme dispersed embryoid bodies at day 17 and day 28 (Fig. 3). Myocardin overexpression from day 10 onwards increased numbers of SMαA^+^ cells versus the Ad-LacZ transduced negative control (16.5% vs 10.5% at day 17 and 33.0% vs 15.1% at d28, Fig. 3A & B). Similarly, the proportion of SMMHC^+^ cells increased with Ad-Myo treatment (2.7% vs 0.6% at d17 and 25.7% vs 3.3% at d28, Fig. 3C & D). The low levels of SMMHC^+^ cells compared to SMαA^+^ cells confirm that at both day 17 and day 28, untreated or Ad-LacZ treated embryoid bodies exhibit SMCs that are relatively immature. However, in addition to increasing the proportion of SMC-like cells in the embryoid body, a major effect of myocardin is to promote maturation of these cells as suggested by the great increase in the proportion of SMMHC^+^ cells by day 28. It should be noted that adenovirus infection by itself resulted in a small increase in SMA^+^ and SMMHC^+^ cells although there was always a clear effect of myocardin over the virally delivered LacZ negative control.

An interesting observation is that although there was a significant increase in SMαA mRNA during normal embryoid body differentiation (Fig. 1C), in the no virus group the proportion of SMαA^+^ cells was not significantly different between day 17 and day 28 (9.3% vs 8.4% respectively, Fig. 3A). This discrepancy may be accounted for by the significantly higher SMαA signal (median signal in the SMαA^+^ group/median signal in SMαA^−^ group) at day 28 relative to day 17 (Fig. 3E). Thus, while the number of SMαA^+^ cells does not change between day 17 and day 28, their SMαA content increases, possibly reflecting increased maturity.

### Myocardin Selectively Regulates CArG-dependent SMC Genes

**Figure 4:**
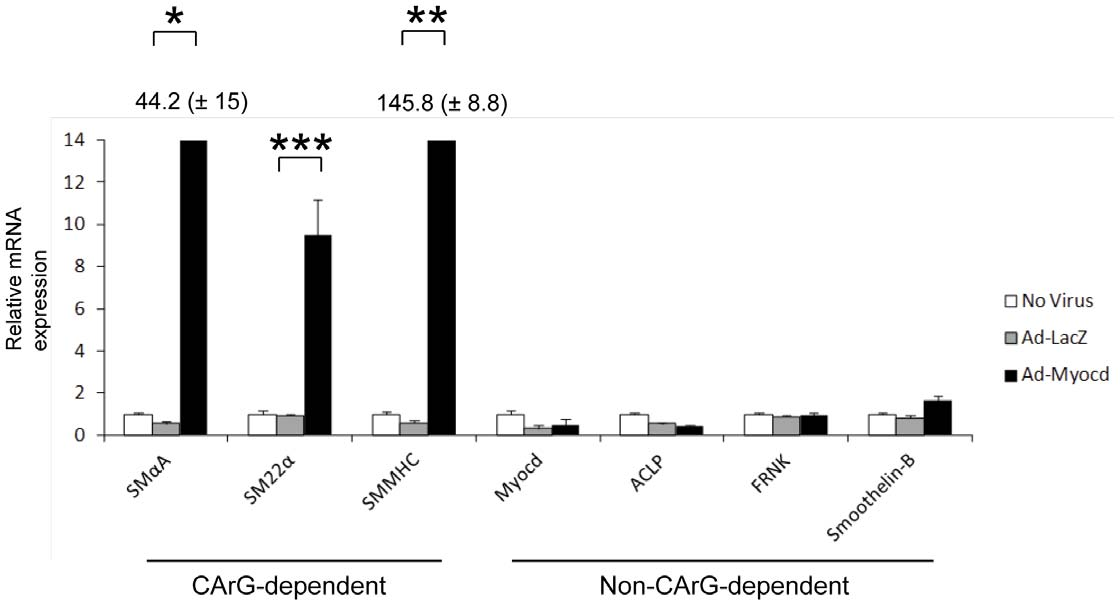
Selective upregulation of CArG-dependent genes by myocardin. Embryoid bodies were treated with no virus, Ad-LacZ or Ad-Myo from day 10 to day 28 and then harvested for RNA. SMC marker expression for a range of CArG-dependent and non-dependent genes was measured using real time RT-PCR and is normalised by three housekeeping genes and then presented relative to no virus controls. RT-PCR data represent means from at least three independent experiments. Error bars represent s.e.m. SMαA and SMMHC expression levels in response to myocardin were significantly higher than the other genes and thus the precise levels are depicted by numbers above the black bars (± s.e.m.).

To examine the specific genes regulated by myocardin in differentiating human embryoid bodies, we carried out quantitative RT-PCR of both CArG-dependent and CArG-independent SMC genes (Fig. 4). Expression of CArG-dependent SMC genes such as SMαA, SM22α and SMMHC was significantly increased by myocardin transduction while non-CArG dependent genes such as smoothelin-B, FRNK and ACLP did not increase. These data demonstrate that myocardin does not activate the full repertoire of SMC genes in cells in differentiating human embryoid bodies.

We also examined expression of markers of different germ layers including nestin & PAX6 (ectoderm), SOX17 & FOXA2 (endoderm), Nkx2–5 & Isl1 (lateral plate mesoderm) and PAX1 & TCF15 (paraxial mesoderm) (Fig. S3A). Viral transduction and myocardin overexpression had minimal effect on ectodermal or endodermal markers. However, mesodermal markers showed increase in expression with Ad-Myo compared to Ad-LacZ transduction, although it appeared that viral transduction itself may have reduced mesodermal marker expression (Ad-LacZ compared to No virus). Nevertheless, while the changes in mesodermal markers with myocardin overexpression were statistically significant, the extent of the changes was greatly reduced compared to those seen in CArG-dependent SMC markers (Fig. 4). Finally, we also examined skeletal muscle and cardiac muscle gene expression. Interestingly, myocardin did not regulate skeletal muscle MHCs (MYH2 and MYH4) but did upregulate the cardiac muscle genes, troponin T and alpha cardiac MHC.

### Increased SMC Contractility with Myocardin Overexpression

**Figure 5:**
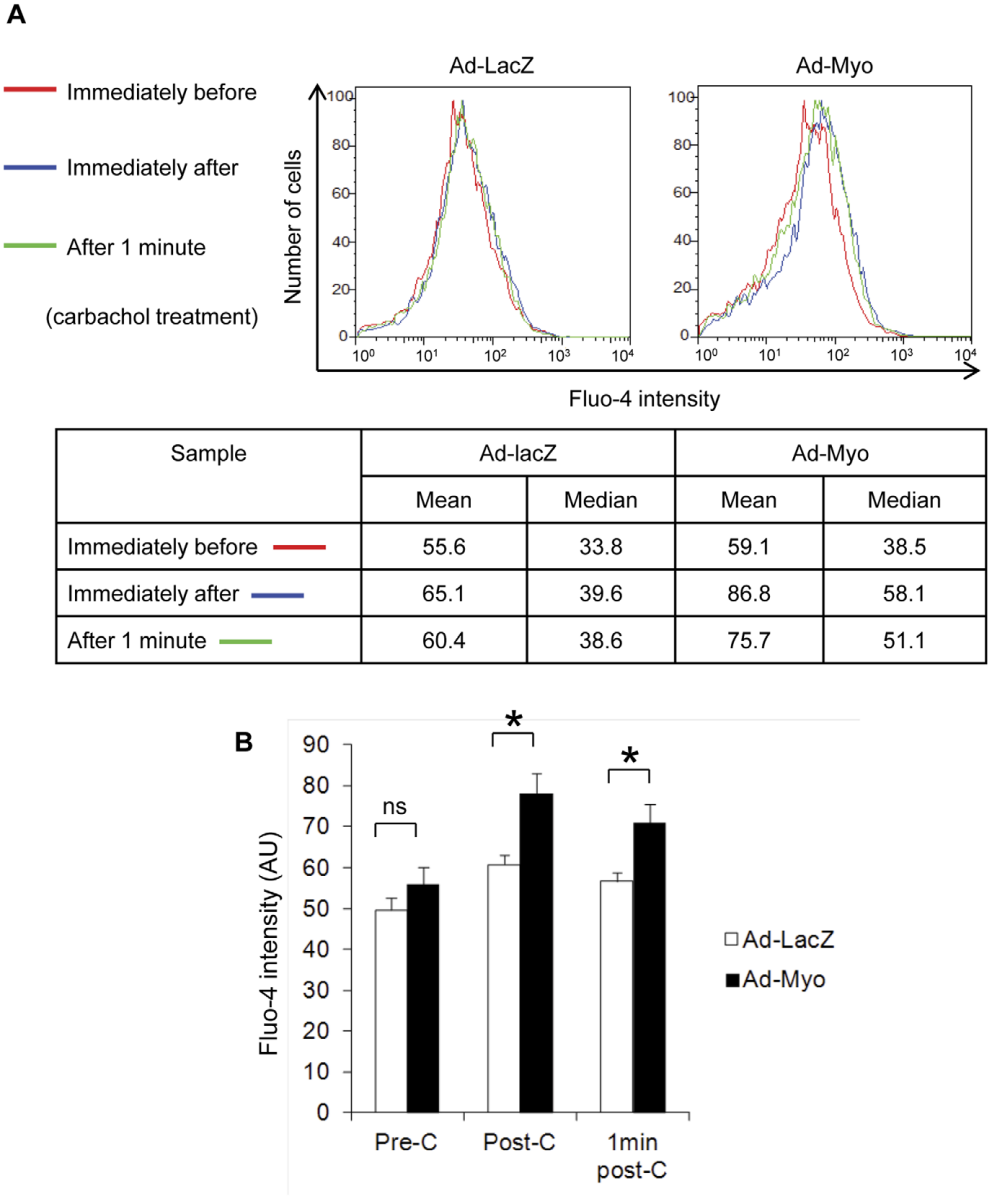
Myocardin promotes calcium influx in response to carbachol. Embryoid bodies were treated with Ad-LacZ or Ad-Myo from day 10 to day 28 and then dispersed into a single cell suspension. Cells were loaded with Fluo-4, a calcium sensitive fluorophore, and intracellular [Ca^2+^] was measured by flow cytometry before and after the addition of the muscarinic agonist, carbachol in arbitrary units (AU). Representative data from a single experiment (A) and means of three studies (B) show significant increases in fluorescence following addition of carbachol. Error bars represent s.e.m., ns = not significant, * = p<0.05, C = carbachol.

We then considered how incomplete activation of SMC genes would impact upon SMC function. We initially examined Ca^2+^ concentration in the embryoid body and the response to a vasoactive agonist, carbachol, as a measure of SMC function and an estimate of contractile potential. Day 28 embryoid bodies were dispersed into single cells and loaded with Fluo-4, a Ca^2+^-sensitive fluorophore. Ca^2+^ influx in response to carbachol was quantified by flow cytometry (Fig. 5). Treatment with Ad-Myo resulted in a small increase in basal levels of intracellular calcium compared to Ad-LacZ treated cells. Moreover, there was a significantly greater increase in intracellular Ca^2+^ in response to carbachol in Ad-Myo treated cells (mean Fluo-4 signal increased from 59.1 pre-carbachol to 86.8 post-carbachol, a 47% increase) in comparison to the Ad-LacZ treated group (55.6 pre-carbachol increased to 65.1 post-carbachol, a 17% increase).

**Figure 6:**
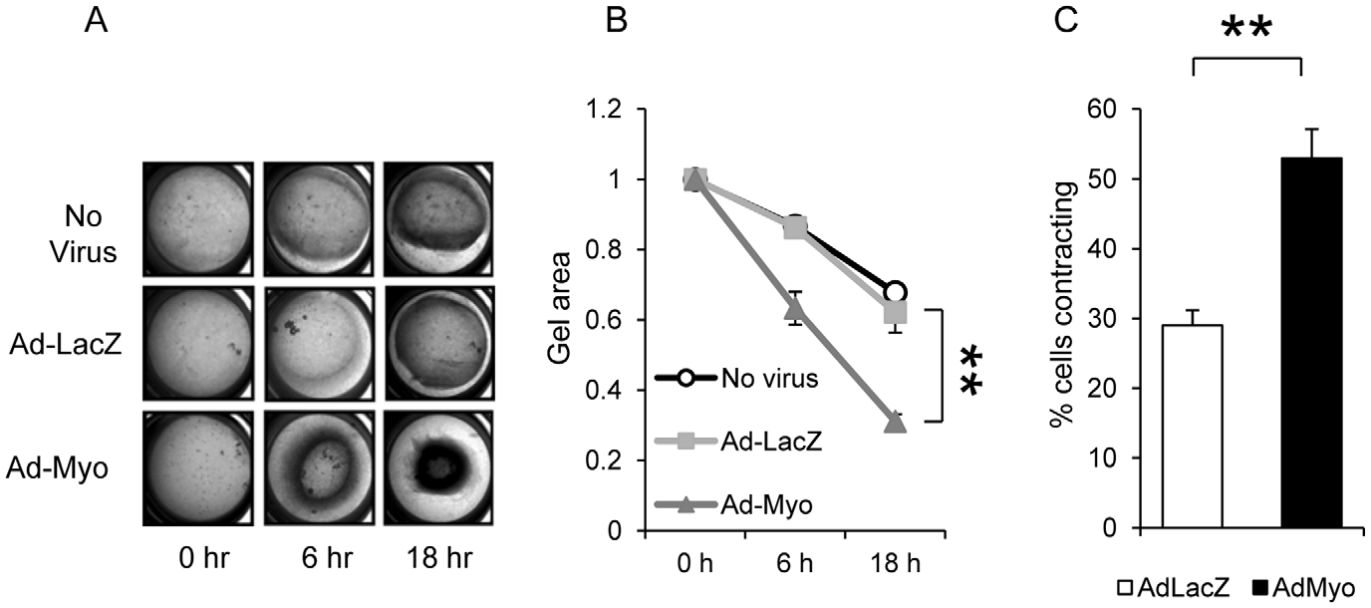
Contractile phenotype is promoted by myocardin overexpression. Embryoid bodies were treated with Ad-LacZ or Ad-Myo from day 10 to day 28, dispersed into a single cell suspension and seeded into collagen gels in 24 well plates (A). Gel contraction in response to carbachol was significantly increased in the Ad-myo group (B). Data points represent the means (±s.e.m.) of three experiments. SMCs derived from human ESCs using a 2-dimensional culture protocol were individually examined for contraction following transduction with Ad-LacZ or Ad-Myo using time lapse microscopy. Percentage of contractile cells increased from 29% with Ad-LacZ to 53% with Ad-Myo (C). Results represent the mean values (±s.e.m.) from 10 randomly chosen optical fields. **p<0.01.

Next, we carried out direct measurements of SMC contractility to assess the effects of myocardin overexpression. Day 28 embryoid bodies were enzymatically dispersed and the cells reseeded in a collagen gel (Fig. 6). Gel contraction in response to carbachol stimulation was significantly increased in the Ad-Myo treated group (63% of starting area at 6 h and 31% at 18 h) compared to either the Ad-LacZ group (86% and 62%) or the no virus group (87% and 68%) (Fig. 6 A & B). In order to observe SMC contraction at the individual cell level, we used an alternative method of differentiating SMCs from hESCs using defined growth factor combinations that we have recently described [17]. Differentiating SMCs having undergone 12 days of PDGF-BB and TGF-β1 treatment were treated with Ad-Myo or Ad-LacZ. After 48 h, the proportion of SMCs contracting in response to carbachol increased from 29% in the Ad-LacZ group to 53% in the Ad-Myo treated group (p<0.01) (Fig. 6C and Fig. S4A). In addition, there was a greater extent of individual cell contraction in the myocardin treated group (20% reduction in contractile cell surface area in the Ad-Myo treated group vs 7% with Ad-LacZ (p<0.01) (Fig. S4B).

Together, these data make a compelling case that myocardin overexpression increases hESC-derived SMC contractility. Moreover, myocardin is able to promote a phenotype that displays functional properties consistent with a more mature contractile phenotype without activating the entire repertoire of SMC genes.

### Myocardin has an Early Dominant Effect in Developing Cells

**Figure 7:**
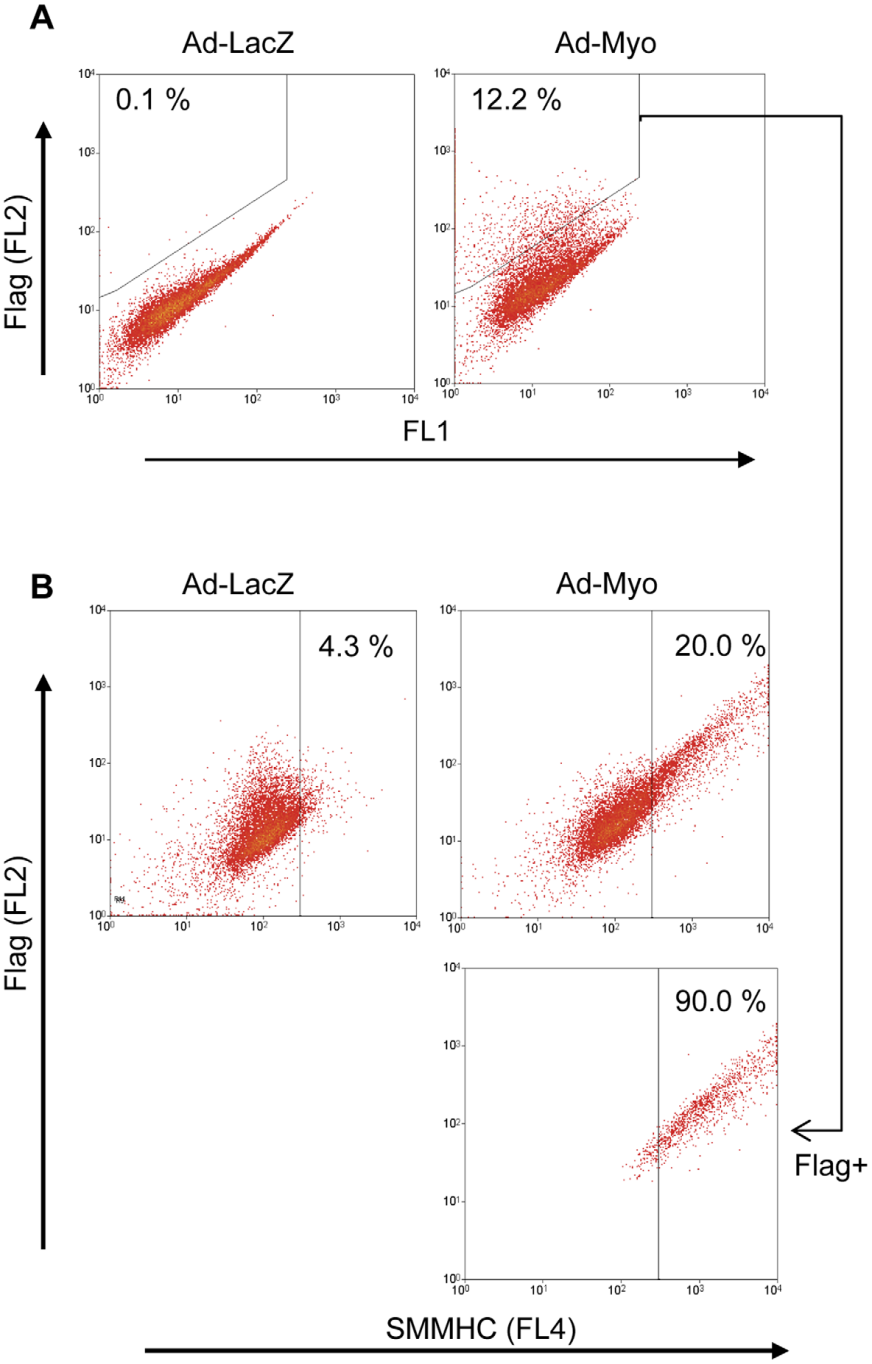
Myocardin has a dominant instructive role on development of SMC-like cells in the embryoid body. Embryoid bodies were treated with Ad-LacZ or Ad-Myo from day 10 to day 28 then dispersed and fixed for flow cytometry. (A) The subset of cells transduced with the Ad-Myo virus was identified by flow cytometric detection of the 3′ FLAG tag fused to the myocardin transgene. (B) The effect of Ad-Myo treatment on %SMMHC^+^ cells was analysed by flow cytometry. The majority (90%) of FLAG^+^ Ad-Myo transduced cells demonstrated a SMC-like phenotype. Data are representative of three independent experiments.

An intriguing question raised by our studies is whether myocardin expression promotes the SMC phenotype in all developing cells, ie affects lineage commitment or whether myocardin simply promotes SMC maturation and contractility in cells that are already committed to the SMC phenotype. Our flow cytometry data show that even in the Ad-Myo treated group, only a minority of cells ultimately expressed SMC markers whilst a large proportion remained SMαA and SMMHC negative. To determine whether these SMC marker-negative cells were a result of low Ad-Myo transduction efficiency or the inability of myocardin to promote the SMC lineage in all developing cells, we carried out flow cytometry with co-labelling for the FLAG tag on the myocardin transgene and SMMHC (Fig. 7). The low proportion of day 28 cells (12%) that were FLAG^+^ implies that transduction efficiency was still a limitation on the ability to generate SMC-like cells. Interestingly, flow cytometric analysis of less well differentiated embryoid bodies (day 21) suggested that transduction efficiency and myocardin expression were higher at earlier stages of differentiation (16.6% FLAG^+^), likely due to greater viral access to cells in smaller less compact embryoid bodies (Fig. S5). In any case, the majority (88–90%) of transduced, FLAG expressing cells also expressed the specific SMC marker SMMHC, suggesting that myocardin has a dominant role in SMC lineage commitment.

**Figure 8:**
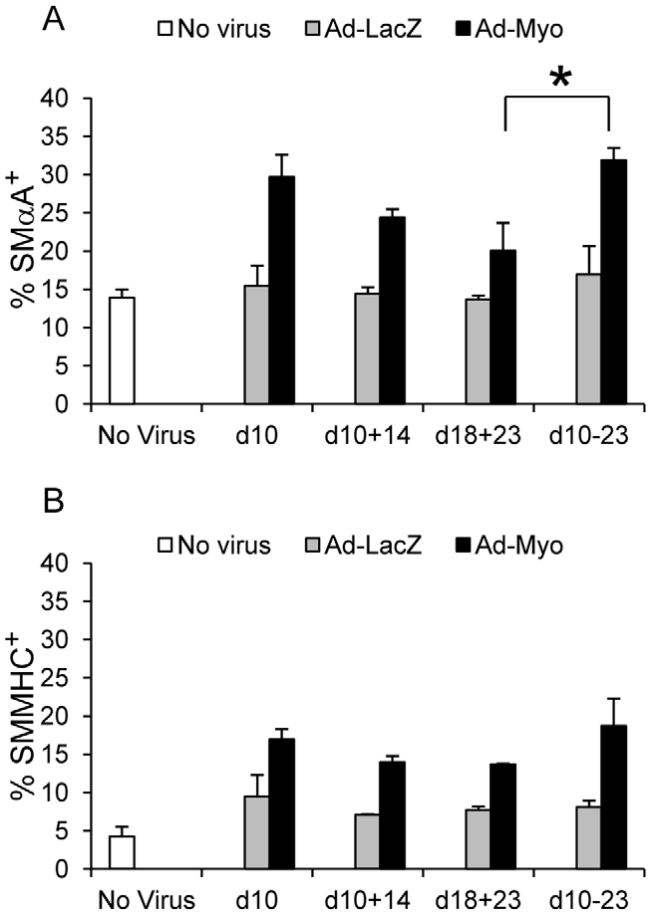
Timing of viral transduction reveals an early role in SMC induction for myocardin. Embryoid bodies were transduced with Ad-LacZ or Ad-Myo early (day 10 alone or days 10 & 14), late (days 18 & 23) or throughout differentiation (days 10, 14, 18 &13). Late delivery of Ad-Myo reduced number of SMαA^+^ cells (A) (*p<0.05 by ANOVA and Tukey HSD) but had no significant effect on SMMHC^+^ cell numbers (B). Values represent mean cell proportions from three independent experiments (± s.e.m.). *p<0.05.

We also investigated whether timing of adenoviral delivery was important and compared induction of SMC marker positive populations by flow cytometry when embryoid body transduction was carried out early (day 10 or days 10 & 14) or late (days 18 & 23) or throughout differentiation (days 10, 14, 18 & 23) (Fig. 8). We found that the number of SMαA^+^ cells was significantly reduced in the late transduction group (days 18 & 23) compared to transduction throughout differentiation (p<0.05). However, early transduction alone (day 10 or days 10 & 14) was not detrimental to SMC induction and generated comparable SMαA^+^ cells to transduction throughout. Interestingly, SMMHC which is a late SMC marker and as such correlates well with maturation was not significantly different regardless of timing of viral transduction. These data are consistent with a role for myocardin in SMC induction or lineage determination early on in development alongside a role in promoting a mature SMC in later development.

## Discussion

We have demonstrated that myocardin overexpression in human embryoid bodies upregulates the subset of SMC genes that are CArG-dependent and is not able to activate the full SMC developmental program. These results extend the findings of Yoshida and colleagues [11] to SMC development in a human system in which contractile SMCs normally develop. Thus although non-CArG dependent genes such as smoothelin or ACLP are expressed in the embryoid body system, there is no increase in their expression in response to myocardin overexpression in contrast to the dramatic increase in expression of CArG-dependent genes such as SMαA, SM22α and SMMHC. In contrast, Long and colleagues [13] reported that myocardin promoted the development of SMC-like cells when overexpressed in a BC3H1 myogenic cell line. This study only reported on expression of CArG-dependent marker genes and did not investigate changes in non-CArG dependent genes. Thus, although the transduced BC3H1 cells showed contractile responses to KCl, it wasn’t clear whether the cells fully took on a contractile SMC phenotype as specific SMC agonists such as angiotensin II or carbachol were not tested. In our study, although only CArG-dependent genes were activated, myocardin overexpression did result in greater Ca^2+^ currents in response to carbachol and increased SMC contraction suggesting that myocardin did indeed promote a more mature contractile SMC phenotype.

The SMCs that develop using the described embryoid body protocol, in the absence of exogenous myocardin, appear to be relatively immature given the low expression of SMMHC and the lack of spontaneous SMC-like contraction by day 28. Other groups have reported higher efficiencies of SMC induction from human ESCs, for example Huang and colleagues [18] reported highly efficient induction of contractile SMCs (94.5% SMαA^+^ cells) using retinoic acid induction on monolayer cultures. However the high concentration of retinoic acid (10 µM) used by Huang and colleagues raises questions as to the physiological nature of the subsequent SMC induction. Indeed, one of the advantages of our embryoid body approach is that it is thought to replicate some aspects of embryonic development such as the generation of cells corresponding to the embryonic germ layers and heterotypic cell-cell and cell-matrix interactions, and potentially offers a more ‘physiological’ system for investigating development [12]. Moreover, while SMC maturation could be accelerated by further manipulation of the culture conditions, the current protocol is entirely suitable for the purposes of a myocardin gain of function study.

When assessing SMC development and identity, in addition to a range of SMC markers it is essential to measure SMC function. Thus, a particular strength of this study is that the effects of myocardin on hESC-derived SMC contractile function have been evaluated in a variety of different ways. We chose to examine Ca^2+^ influx secondary to vasoactive drugs as this is usually a prerequisite for SMC contraction and also directly measured contraction in seeded collagen gels. Moreover, we used a novel chemically-defined in vitro system that we have recently described to visualise the contraction of individual SMCs. These multiple lines of evidence strongly suggested that myocardin overexpression promotes a contractile phenotype in human ESC-derived SMCs. This may be of importance in regenerative medicine applications where mature contractile SMCs are required [19].

It is instructive to examine the pattern of gene regulation associated with myocardin overexpression in developing human embryoid bodies. The most striking feature is a dramatic upregulation of CArG-dependent SMC genes, consistent with myocardin’s known mechanism of action through promoting SRF-CArG box interactions [1], [4], [5], [6]. Consistent with previous studies by Yoshida and colleagues [11] and Long and colleagues [13], increased expression of cardiac muscle markers was also seen. Of note, there was no increase in skeletal muscle markers and previous studies have demonstrated that myocardin has a dominant effect favouring SMC induction over skeletal muscle in developing somites [20]. An important question to consider is how is SMC contractility increased when the predominant effect is on the subset of genes that are CArG-dependent? A relevant factor may be that other (non-CArG-dependent) genes required for contraction are also being induced in the developing embryoid body. We hypothesise that cells may be ‘primed’ for acquiring contractile function and this process is accelerated by the CArG-dependent upregulation of SMC contractile genes. It should be noted however that some non-CArG-dependent SMC genes such as smoothelin B and a number of mesodermal markers also show modest increases in expression and importantly non-CArG-dependent mechanisms of action of myocardin have been previously described [21].

A key question is whether myocardin promotes commitment to the SMC lineage in uncommitted multipotent cells in the embryoid body or whether it acts only to accelerate/promote SMC maturation once precursor cells have already undergone specification. Previous studies have shown that in A404 cells, the expression of at least a low level of myocardin is associated with the ability of these cells to form SMCs efficiently in contrast to the parental P19 line which does not express myocardin and does not form SMC at high frequency [11]. It has also been reported that myocardin expression has a dominant effect on skeletal muscle progenitors [20]. In the present study, although the majority of cells in Ad-Myo treated embryoid bodies did not express SMC markers, co-localisation with FLAG demonstrated that these cells were likely not transduced, possibly due to inefficiencies with viral transduction given the complex layering and compact three-dimensional structure of the embryoid body. In fact, the vast majority of cells that expressed the FLAG tag also expressed the highly specific SMC marker, SMMHC, suggesting that myocardin does indeed have a dominant effect on a wide variety of cells in the developing human embryoid body.

Further analyses on timing of transgene delivery suggest that early transduction is important for SMC lineage determination, with reduced induction of SMαA^+^ cells with late transduction only. In keeping with this, adenoviral delivery timing did not influence numbers of SMMHC^+^ cells, perhaps since this marker reflects maturation better than lineage induction (as immature SMCs may be SMαA^+^ yet SMMHC^−^). It is recognised that a limitation of the experiments presented is the sub-optimal adenoviral transduction of embryoid bodies, likely due to the multilayered nature of these structures, which may dilute the experimental effects of myocardin. One possibility for further experiments would be to generate an inducible myocardin system with lentiviral delivery prior to embryoid body formation, which would result in transgene integration into the host genome and may lead to more effective transgene expression. For the purposes of this study, we have focussed exclusively on myocardin gain of function. Studies in the mouse using myocardin-null ESCs [9], [10] have suggested that myocardin may not be absolutely required for SMC development, possibly due to redundant mechanisms. Further studies in human ESCs using a loss of function approach are indicated to clarify the role of myocardin in human SMC development and determine the basis of any possible redundancy.

In conclusion, these studies demonstrate that myocardin overexpression was able to promote the formation and maturation of SMC-like cells in differentiating human embryoid bodies in a dominant manner despite increased gene expression being predominantly confined to the subset of CArG-dependent genes. These findings have implications for vascular tissue engineering and other applications requiring large numbers of functional SMCs.

## Materials and Methods

### Cell Culture

Human H9 ESCs were obtained from Wicell (Madison, Wisconsin) and cultured on irradiated mouse embryonic fibroblasts (MEFs) using KSR medium [advanced DMEM/F12 (Life Sciences), 20% knockout serum replacer (Life Sciences), 2 mM L-glutamine and 0.1 mM β-mercaptoethanol] supplemented with 4 ng/ml FGF-2 (R&D Systems) or on gelatin coated plates using chemically defined media (CDM) [IMDM and F12 (1∶1 mix), 5 mg/ml bovine serum albumin (BSA, Sigma), 1% lipid concentrate (Life Sciences), 450 µM monothioglycerol (Sigma), 7 µg/ml insulin (Roche) and 15 µg/ml transferrin (Roche)] supplemented with 10 ng/ml Actinic A (R&D Systems) and 12 ng/ml FGF-2. ESC colonies were passaged by a brief treatment with 1 mg/ml collagenase IV (dissolved in advanced DMEM/F12, 20% KSR and 2 mM L-glutamine) and then mechanically scraped using a 5 ml pipette. ESC clumps were washed with PBS and replated or cultured in suspension in embryoid body medium [DMEM high glucose (Life Sciences), 20% foetal bovine serum, 0.1 mM non-essential amino acids, 1 mM pyruvate and 1% penicillin-streptomycin-glutamine (Life Sciences)] to generate embryoid bodies. As previously described for mouse ESCs [16], embryoid bodies were plated onto gelatin coated plates at day 6 to allow attachment and then treated with 10 nM all trans-retinoic acid (atRA, Sigma) from d7-d10. Embryoid bodies were harvested at varying time-points for RNA or dispersed with collagenase and elastase [1 mg/ml collagenase type 2, 0.75 u/ml porcine elastase and 1 mg/ml soya bean trypsin inhibitor (all from Worthington Biochem Corp) in PBS] then fixed in BD Cytofix/Cytoperm solution (BD Biosciences) for 15 min at 4°C prior to flow cytometric analysis.

Two-dimensional human ESC differentiation was carried out as described previously [17]. Briefly, human ESCs were induced to a mesodermal fate using FGF-2, BMP-4 and LY294002 for 36 h then FGF-2 and BMP-4 for a further 3.5 days. Cells were then treated with PDGF-BB and TGF-β1 for 12 days to generate contractile SMCs.

### Adenoviruses

Replication deficient adenoviruses encoding either β-galactosidase (Ad-LacZ) or myocardin (Ad-Myo) were generated using standard methods by the University of Iowa Gene Transfer Vector Core [6], [22] and obtained with the approval of Dr GK Owens (University of Virginia, USA). Embryoid bodies were transduced using the adenoviruses on days 10, 14, 18 and 23 of differentiation. Optimum virus concentration for maximising transduction efficiency was estimated by titrating the Ad-LacZ concentration (Figure S1) and a concentration of 0.5×10^7^ pfu/ml was used for these studies and applied overnight.

### Real Time RTPCR

RNA was extracted using Trizol (Life Sciences) according to the manufacturer’s instructions and cDNA was synthesised using the Superscript III kit (Life Sciences). Quantitative PCR was carried out using the SYBR Green PCR Master Mix (Applied Biosystems) in a Rotor Gene 6000 (Corbett). A full list of primers can be found in table 1. A reference standard curve was included with every run and all genes were normalised to the geometric mean of three reference genes [23]: glyceraldehydes-3-phosphate dehydrogenase (GAPDH), ubiquitin C (UBC) and 18S. Data in bar graphs are presented as mean ± s.e.m. of 3 independent experiments.

**Table 1:** Primer sequences used for real time RT-PCR.

Gene	Forward primer	Reverse primer	Annealing Temp (°C)
SMαA	CACTGTCAGGAATCCTGTGA	CAAAGCCGGCCTTACAGA	65
SM22α	TCTTTGAAGGCAAAGACATGG	TTATGCTCCTGCGCTTTCTT	65
SMMHC	AGATGGTTCTGAGGAGGAAACG	AAAACTGTAGAAAGTTGCTTATTCACT	65
Myocardin	TGCAGCTCCAAATCCTCAGC	TCAGTGGCGTTGAAGAAGAGTT	60
Smoothelin-B	TGCCGAGGCCCAGTGCCTTA	GGGTCCAGCCACGTCTGCTC	60
ACLP	GGCATCGTCAACGGGGCCAA	GCGGTGCACCTGCTCCATGA	60
FRNK	TCGGCTTGGCCCTGAGGACA	GGCGTGAGCAGCAGTCAGCA	60
cTnT	GGAAGAGGCAGACTGAGCGGGA	TCCCGCGGGTCTTGGAGACTT	60
UBC	ATTTGGGTCGCGGTTCTTG	TGCCTTGACATTCTCGATGGT	60
GAPDH	AACAGCCTCAAGATCATCAGC	GGATGATGTTCTGGAGAGCC	60
18S	GACACGGACAGGATTGACAGATTG	TGCCAGAGTCTCGTTCGTTATCG	60
Nestin	GAAACAGCCATAGAGGGCAAA	TGGTTTTCCAGAGTCTTCAGTGA	60
NKX2.5	CAAGTGTGCGTCTGCCTTT	CAGCTCTTTCTTTTCGGCTCTA	60
ISL1	AGATTATATCAGGTTGTACGGGATCA	ACACAGCGGAAACACTCGAT	60
FoxA2	GGGAGCGGTGAAGATGGA	TCATGTTGCTCACGGAGGAGTA	60
Sox17	CGCACGGAATTTGAACAGTA	GGATCAGGGACCTGTCACAC	60
PAX1	CACACTCGGTCAGCAACATC	GGTTTCTCTAGCCCATTCACTG	60
TCF15	GCACCTTCTGCCTCAGCAACCAGC	GGTCCCCCGGTCCCTACACAA	60
MYH2	AGCTCCAAGAACTGTCTCACTCCCA	TGGGCCTCAATGCGCTCCCT	60
MYH4	TGTCTGCTTTGAGCCTGCCACC	TTACAGTAGCTCCAGCTTCGGTCTT	60

### Immunofluorescence and x-gal Staining

Embryoid bodies were fixed in 4% paraformaldehyde and then permeabilised with 0.2% triton in PBS. Specimens were blocked with 3% BSA and then the following primary antibodies were used: anti-SMαA (Sigma, A2547) at 1∶1000 and anti-SMMHC (Sigma, M7786) at 1∶2000. Anti-mouse or anti-rabbit secondaries labelled with Alexa Fluor 568 (Life Sciences) were used at 1∶400 and nuclei were counterstained with 0.5 µg/ml 4′,6-diamidino-2-phenylindole (DAPI). For x-gal staining, paraformaldehyde fixed embryoid bodies were incubated overnight at 37°C with x-gal staining solution (1 mg/ml x-gal, 5 mM potassium ferricyanide, 5 mM potassium ferrocyanide and 2 mM magnesium chloride in PBS). Images were taken using a Zeiss Axiovert 200 M microscope.

### Flow Cytometry and Intracellular Calcium

Fixed single cell suspensions were blocked with 3% BSA and then labelled with primary antibodies in BD Perm/Wash buffer (BD Biosciences) as follows: anti-SMαA-FITC (Sigma, F3777) at 1∶1000, anti-SMMHC (Sigma, M7786) at 1∶500 and anti-FLAG-PE (Abcam, ab72469) at 1∶300. Anti-mouse-PE or anti-mouse Alexa Fluor 647 secondaries (Life Sciences, 1∶400) were used for anti-SMMHC detection. Samples were analysed on a Cyan ADP analyzer (Beckman Coulter). Both FL1 and FL2 channels were measured for all samples to distinguish specific signal for SMαA (FL1 in Fig. 3A) and SMMHC (FL2 in Fig. 3B) due to the high levels of autofluorescence in embryoid body-derived cells. For the calcium studies, an unfixed single cell suspension was isolated from embryoid bodies as described above and loaded with 2.5 µM Fluo-4 (Molecular Probes), a calcium sensitive fluorophore, for 1 hr at 37°C. Calcium influx was induced by the addition of 100 µM carbachol (Sigma) and cellular [Ca^2+^] was estimated by fluorescence in the FL1 channel of the flow cytometer immediately prior to, immediately post- and 1 min post- addition of carbachol. Data presented are representative of 3 independent experiments.

### Western Blot Analysis

Proteins were extracted using lysis buffer (10 mM Tris-HCl, pH 7.6, 140 mM NaCl, 1 mM EDTA, 1% Triton X-100, pro­tease and phosphatase inhibitor cocktail (Sigma)), and concentrations determined using a BCA Protein Assay Kit (Thermo Scientific). Samples were separated by SDS-PAGE and proteins transferred on to polyvinylidene difluoride membranes, blocked in 5% milk in Tris-buffered saline and 0.05% Tween 20, incubated with primary antibodies against myocardin (Sigma M8948; 1∶1000 dilution) and β-actin (Sigma A2228; 1∶10,000 dilution) followed by incubation with horseradish peroxidase-conjugated secondary antibodies (Dako). Signals were detected using ECL Western Blotting Detection Reagents (GE Healthcare Life Sciences).

### Contraction Assays

The collagen gel was prepared by mixing 8 parts of ice-cold collagen Type-1 solution (Sigma Aldrich, UK) with 1 part of 10× PBS. The pH of the mixture was adjusted to 7.2–7.4. The cells were resuspended at a density of 0.6×106 cells/ml of collagen mixture and 500 µl was pipetted into each well of a 24-well plate. Gels were polymerised at 37°C 45–60 min. The contraction assay was initiated by the addition of 50 µM of carbachol. The gel areas were measured at 0, 6 & 18 hours of carbachol stimulation using Image J software.

For assessment of individual cell contraction, SMCs were generated using the two-dimensional directed differentiation protocol. SMCs were transduced with Ad-LacZ or Ad-Myo and examined for contractile responses by time-lapse microscopy 48 h after viral transduction in response to 50 µM carbachol.

### Statistics

Means between two groups were compared using the student’s t-test. Multiple groups were compared using one way ANOVA and differences between groups confirmed by Tukey HSD test.

## Supporting Information

Figure S1 **Optimisation of adenoviral titres for transduction.** Embryoid bodies were transduced using a range of Ad-LacZ titres (0.5×10^6^ to 1.5×10^7^ pfu/ml) and then stained with X-gal to identify optimal viral titres to ensure maximal transduction. Bar = 100 µm.
(PDF)

Figure S2 **Myocardin overexpression promotes a contractile SMC appearance in embryoid bodies.** Effects of Ad-LacZ (A and C) or Ad-Myo (B and D) treatment from day 10 to day 28 on the appearance of cells in the embryoid body outgrowth. Phase contrast images were taken at day 28. Bar = 100 µm.
(PDF)

Figure S3 **Mesoderm and cardiac genes are upregulated by myocardin overexpression.** Embryoid bodies were treated with no virus, Ad-LacZ or Ad-Myo from day 10 to day 28 and then harvested for RNA. Real time RT-PCR was used to quantify markers of ectoderm, endoderm and mesoderm (A). Markers of skeletal muscle (B) and cardiac muscle (C) were also investigated. RT-PCR data represent means from three independent experiments (± s.e.m.). *p<0.05, **p<0.01.
(PDF)

Figure S4 **Myocardin overexpression increases human ESC-derived SMC contractility.** Human ESCs were induced to undergo differentiation on a 2-dimensional surface using defined growth factors. Two days after Ad-LacZ or Ad-Myo transduction, individual SMCs were visualised at 0 min and 15 min for contractile activity in response to carbachol stimulation. The percentage change of cell surface area was determined from 20 contracting cells selected randomly from 10 different optical fields. (***p<0.001).
(PDF)

Figure S5 **Reduced myocardin-FLAG transgene expression with embryoid body maturation.** Embryoid bodies were treated with Ad-LacZ or Ad-Myo from day 10 onwards and dispersed and fixed for flow cytometry at day 21 (A–C) or day 28 (D–F). The subset of cells transduced with the Ad-Myo virus was identified by flow cytometric detection of the 3′ FLAG tag fused to the myocardin transgene, which was higher at day 21 than day 28. The effect of myocardin-FLAG transgene expression on %SMMHC^+^ cells was analysed by flow cytometry. The majority (88–89%) of FLAG^+^ Ad-Myo transduced cells demonstrated a SMC-like phenotype.
(PDF)
