# Supplementary figures and images for: Myocardin Overexpression Is Sufficient for Promoting the Development of a Mature Smooth Muscle Cell-Like Phenotype from Human Embryonic Stem Cells
Source: PLoS One. 2012 Aug 28;7(8):e44052. doi: 10.1371/journal.pone.0044052 (PMC3429416; doi:10.1371/journal.pone.0044052)

## Supplementary Figure S1

$0.5 \times 10^6/\text{ml}$

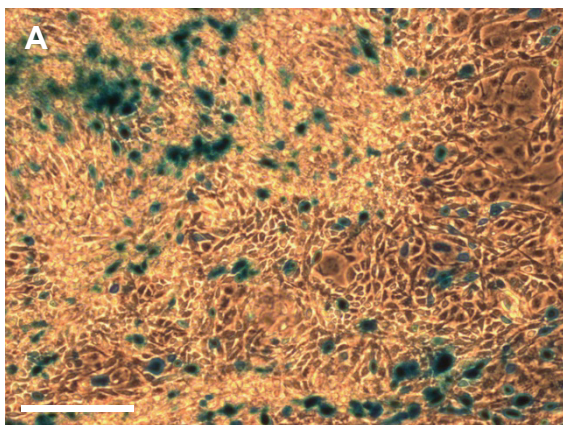

$1.5 \times 10^6/\text{ml}$

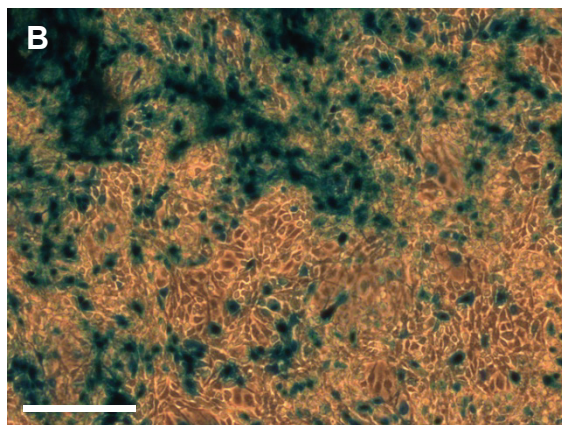

$0.5 \times 10^7/\text{ml}$

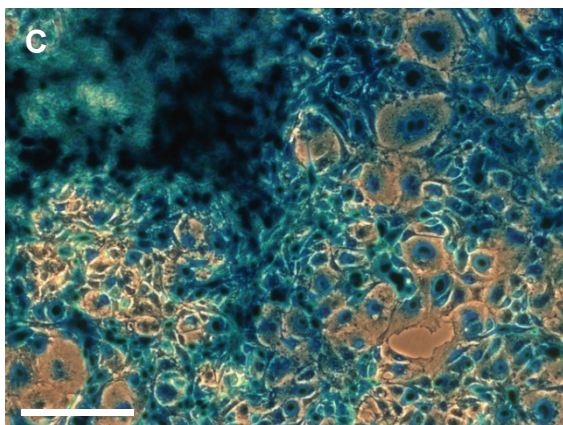

$1.5 \times 10^7/\text{ml}$

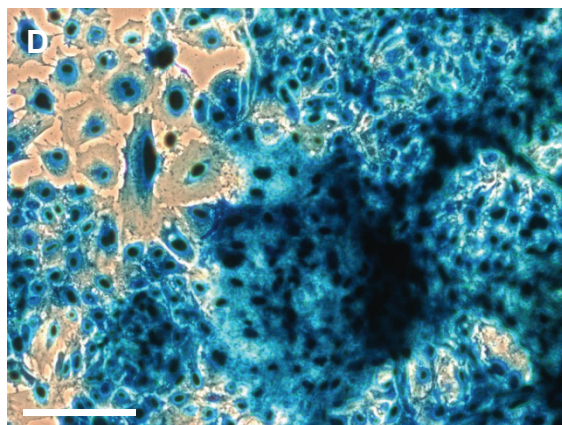

Supplement: Figure S1 — Optimisation of adenoviral titres for transduction. Embryoid bodies were transduced using a range of Ad-LacZ titres (0.5×10^6^ to 1.5×10^7^ pfu/ml) and then stained with X-gal to identify optimal viral titres to ensure maximal transduction. Bar = 100 µm. (PDF) [file pone.0044052.s001.pdf]

Supplementary Figure S2

Ad-LacZ

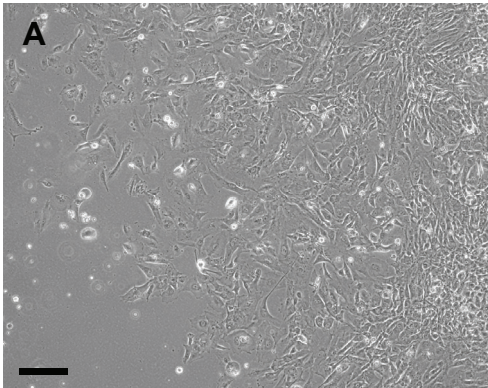

Ad-Myo

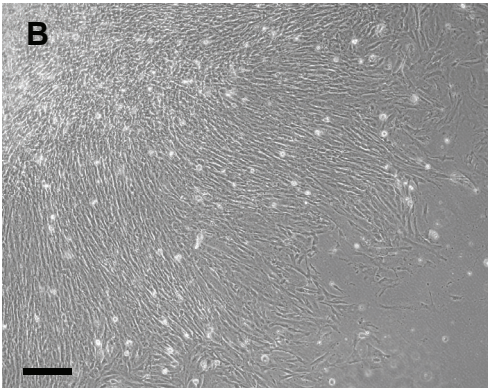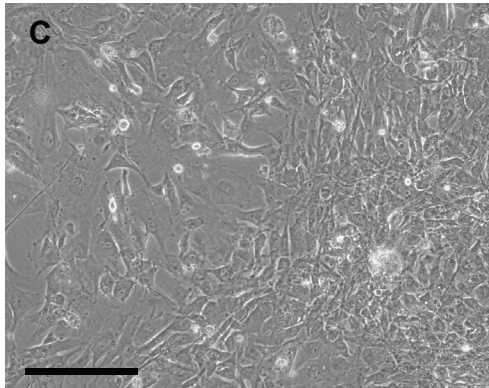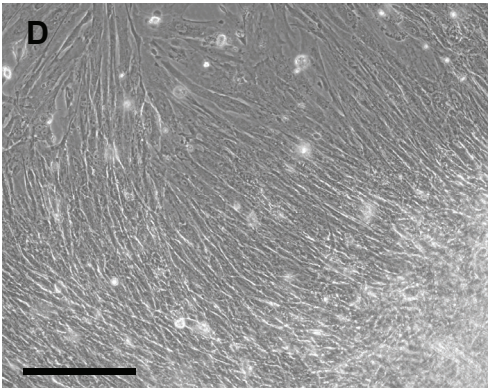

Supplement: Figure S2 — Myocardin overexpression promotes a contractile SMC appearance in embryoid bodies. Effects of Ad-LacZ (A and C) or Ad-Myo (B and D) treatment from day 10 to day 28 on the appearance of cells in the embryoid body outgrowth. Phase contrast images were taken at day 28. Bar = 100 µm. (PDF) [file pone.0044052.s002.pdf]

Supplementary Figure S3

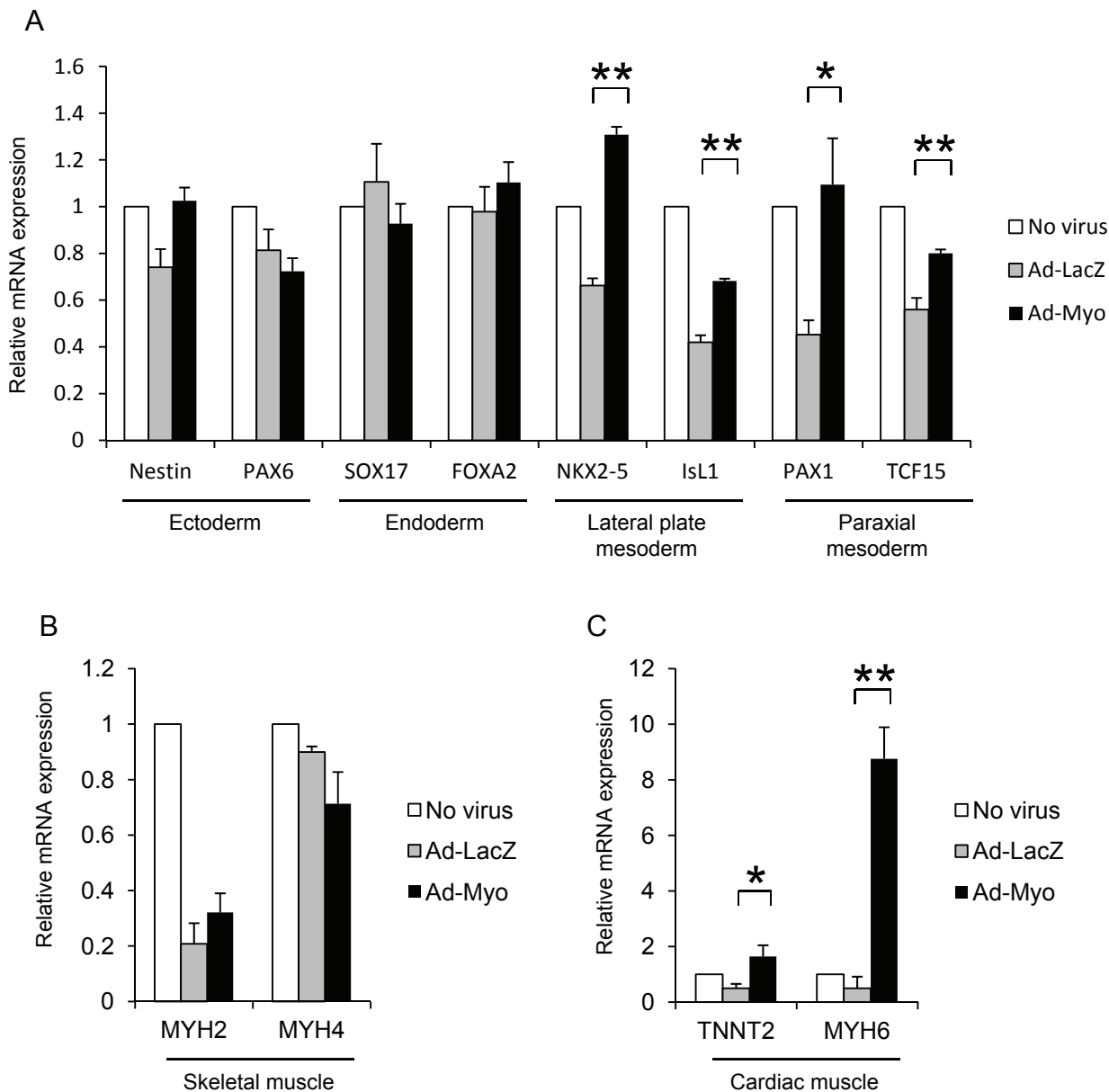

Supplement: Figure S3 — Mesoderm and cardiac genes are upregulated by myocardin overexpression. Embryoid bodies were treated with no virus, Ad-LacZ or Ad-Myo from day 10 to day 28 and then harvested for RNA. Real time RT-PCR was used to quantify markers of ectoderm, endoderm and mesoderm (A). Markers of skeletal muscle (B) and cardiac muscle (C) were also investigated. RT-PCR data represent means from three independent experiments (± s.e.m.). *p<0.05, **p<0.01. (PDF) [file pone.0044052.s003.pdf]

Supplementary Figure S4

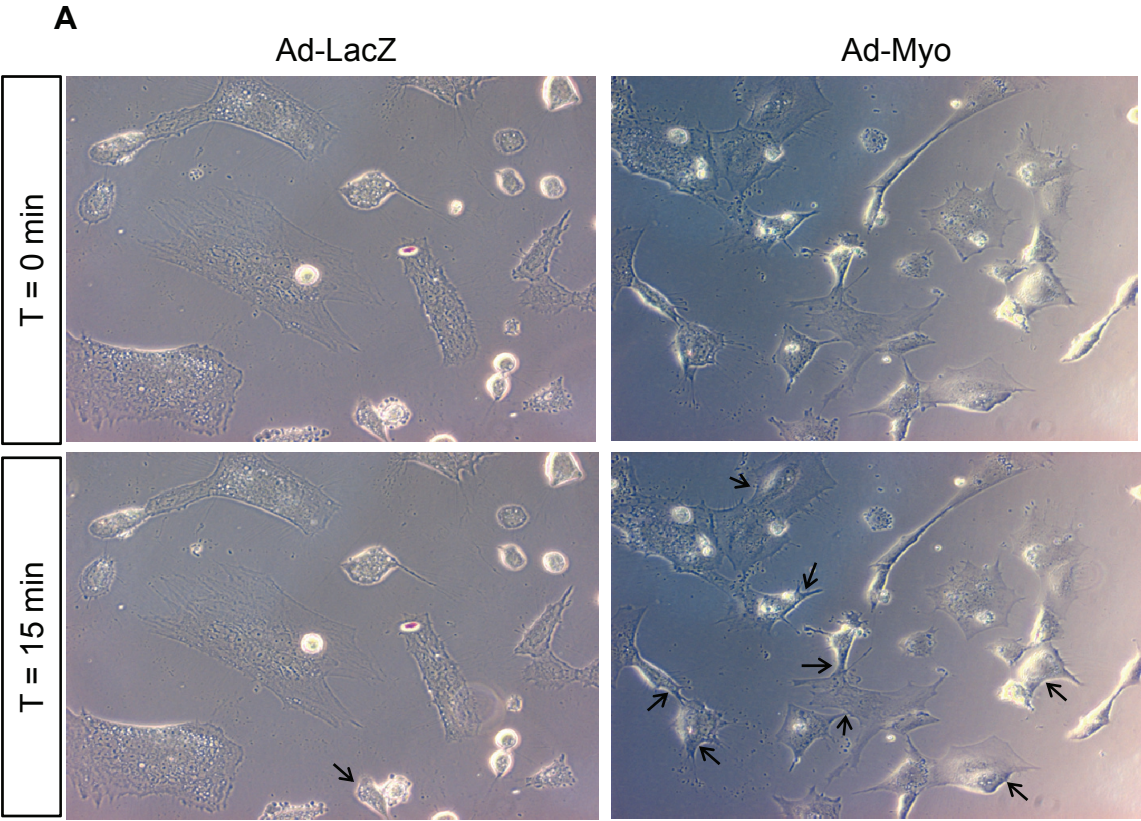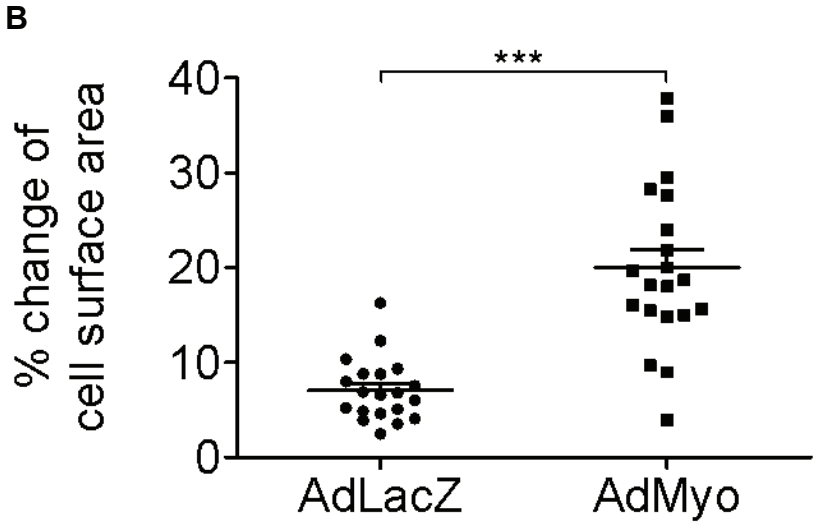

Supplement: Figure S4 — Myocardin overexpression increases human ESC-derived SMC contractility. Human ESCs were induced to undergo differentiation on a 2-dimensional surface using defined growth factors. Two days after Ad-LacZ or Ad-Myo transduction, individual SMCs were visualised at 0 min and 15 min for contractile activity in response to carbachol stimulation. The percentage change of cell surface area was determined from 20 contracting cells selected randomly from 10 different optical fields. (***p<0.001). (PDF) [file pone.0044052.s004.pdf]

Supplementary Figure S5

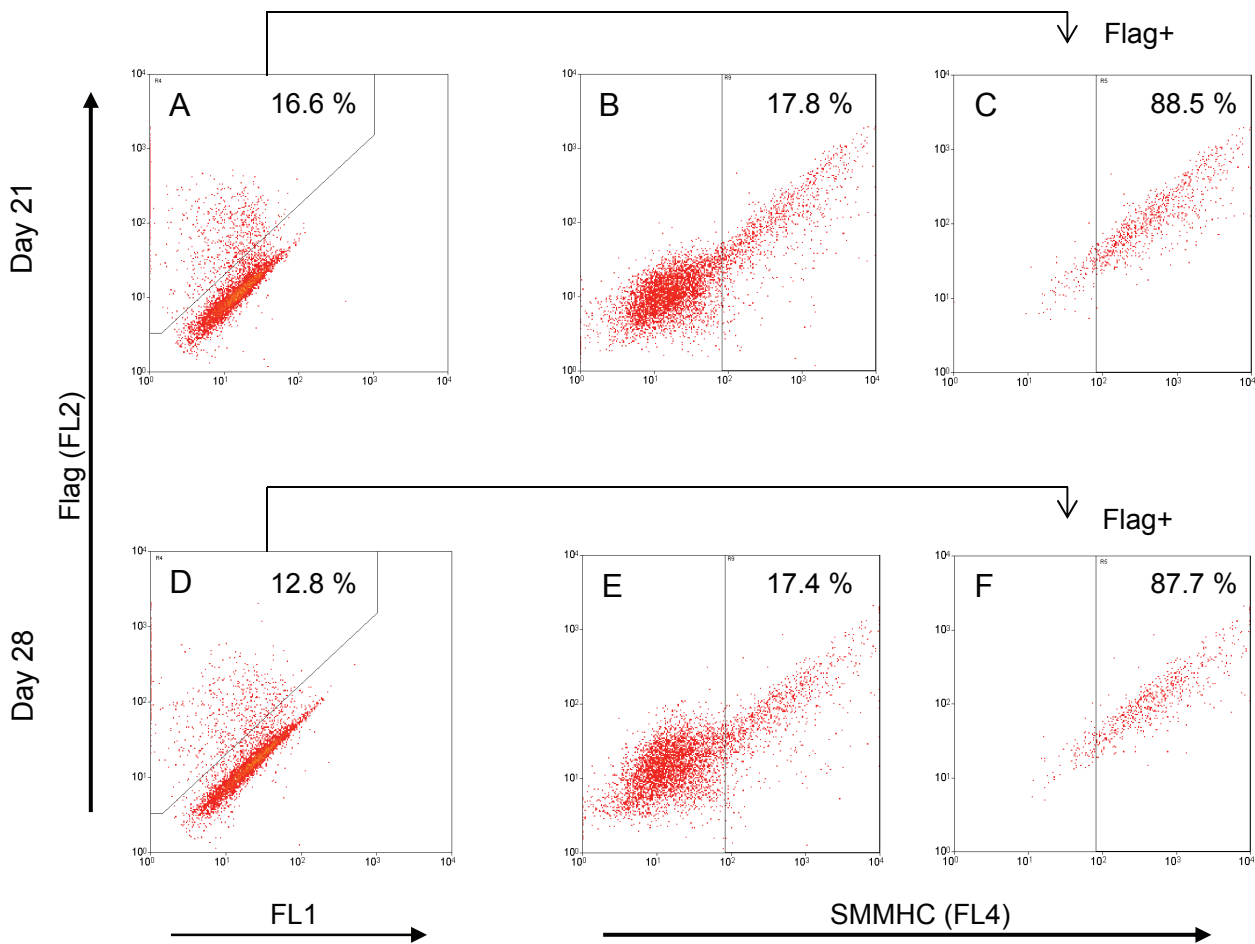

Supplement: Figure S5 — Reduced myocardin-FLAG transgene expression with embryoid body maturation. Embryoid bodies were treated with Ad-LacZ or Ad-Myo from day 10 onwards and dispersed and fixed for flow cytometry at day 21 (A–C) or day 28 (D–F). The subset of cells transduced with the Ad-Myo virus was identified by flow cytometric detection of the 3′ FLAG tag fused to the myocardin transgene, which was higher at day 21 than day 28. The effect of myocardin-FLAG transgene expression on %SMMHC+ cells was analysed by flow cytometry. The majority (88–89%) of FLAG+ Ad-Myo transduced cells demonstrated a SMC-like phenotype. (PDF) [file pone.0044052.s005.pdf]
